# Evaluating Stress-Mediated Microbial Pathogenesis in Golden Shiners, *Notemigonus crysoleucas*

**DOI:** 10.3389/fphys.2022.886480

**Published:** 2022-05-11

**Authors:** Sindhu Kaimal, Bradley D. Farmer, Nilima N. Renukdas, Hisham A. Abdelrahman, Anita M. Kelly

**Affiliations:** ^1^ Department of Aquaculture and Fisheries, The University of Arkansas at Pine Bluff, Pine Bluff, AR, United States; ^2^ United States Department of Agriculture, Agriculture Research Service, Harry K. Dupree Stuttgart National Aquaculture Research Center, Stuttgart, AR, United States; ^3^ Department of Veterinary Hygiene and Management, Faculty of Veterinary Medicine, Cairo University, Giza, Egypt; ^4^ Alabama Fish Farming Center, Auburn University, Greensboro, AL, United States

**Keywords:** Golden Shiner, flow cytometry, stress, water temperature, columnaris disease, *Flavobacterium covae*

## Abstract

*Flavobacterium covae* (columnaris) is a microbial pathogen of the Golden Shiner (*Notemigonus crysoleucas*), a principal bait species. We investigated the effects of density and water temperature on the survival of fish subjected to a columnaris challenge and whether flow cytometry (FCM) could be a fast and reliable method to distinguish and enumerate *F. covae* populations from water and fish in experimental tanks. Juvenile Golden Shiners averaging 2.62 (±0.78 S.D.) g (negative for *F. covae*) were used in simultaneous trials at 22°C and 28°C in two ultra-low flow-through systems: each consisting of four treatments and five replicates per treatment. Treatments were fish stocked at either 600 fish/m^3^ or 2,400 fish/m^3^ and either challenged with *F. covae* or not; survival was observed for 48 h after challenge. Samples of water and fish tissue were obtained for FCM enumerations and validation by qPCR. No significant differences in survival were recorded between density treatments; however, high temperature and columnaris challenge treatments showed significantly higher mortality. Bacterial enumeration (number/mL) by FCM highly correlated with bacterial counts *r* = 0.81 (*p* = 0.001) in the water samples. Higher water temperatures may have increased columnaris infections and mortality in Golden Shiners. Flow cytometry is a reliable method of enumerating *F. covae* from experimental tank water samples.

## Introduction

*Flavobacterium covae* (previously *F. columnare* genomovar II; LaFrentz et al., 2022), a pathogenic, gram-negative bacterium, is the causative agent of columnaris disease in wild and cultured fish (see review by Loch and Faisal 2015). Although *F. covae* can act as a primary pathogen (Plumb 1999; Soto et al., 2008), it is considered a secondary or an opportunistic pathogen, causing heavy mortalities in stressed fish (Durborow et al., 1998).

Intensive aquaculture practices include rearing fish in high densities (Wedemeyer 1996), which can impose a chronic stressful environment. Stressful environments result in suppressed immunocompetence and a compromised ability to defend against pathogens like *F. covae,* a ubiquitous pathogen in aquatic environments (Durborow et al., 1998). Further exposure to acute stressors such as fluctuations in water temperature (Durborow et al., 1998) or handling during transportation can result in sudden disease outbreaks (Wakabayashi and Egusa 1972; Kumar et al., 1986; Wakabayashi 1991).

Infections caused by *F. covae* may result in skin lesions, fin erosion, and gill necrosis. As the disease progresses, more organs are infected, ultimately resulting in mortality. *F. covae* is identified by characteristic long, slender rod-shaped bodies without a flagellum. Strains vary from 0.3 to 0.7 μm in width and 3–10 μm in length. In selective agar, they form flat, yellow, rhizoid, and strongly adherent colonies and spread across the surface, forming irregular margins. Scrapings from infected gill tissues show bacterial cells forming characteristic columnar lumps known as “haystacks.” Columnaris is known by several names such as “saddleback”, “fin rot”, or “cotton wool disease” based on where the infection forms. As the infection proceeds, external tissues, especially the gills, show necrosis and noticeable damage (Davis 1922; Beck et al., 2012).

Although morphologically indistinguishable, four species of *F. covae* have been identified (LaFrentz et al., 2022). Not all species of the bacterium are equally virulent (Shoemaker et al., 2008; LaFrentz et al., 2012). A strain is considered highly virulent if it kills the entire population of fish within a 24 h period and less virulent if 100% mortality is achieved only 96 h after exposure (Thomas-Jinu and Goodwin 2004; Declercq et al., 2013).

Quick detection of bacteria is essential for disease prevention and treatment. To determine the possible severity of an infection, it may be necessary to detect bacteria from fish and the surrounding water. Enumerations help assess the infection status, determine control measures, and whether the control measures used have been effective. Traditionally, fish health professionals use the plate count method for bacterial enumerations. This method is time-consuming, requiring at least 24–48 h for colonies to develop, an essential factor when treating a potential outbreak on a commercial farm. An additional concern is most media used in the bacterial plates are not exclusive to *F. covae*; often, other bacteria associated with the fish and the environment are also cultured with the target bacteria making counts inaccurate. More recent methods of microbial pathogen identification and enumeration include immunofluorescence antibody technique (IFAT), quantitative polymerase chain reaction (qPCR; Gibbs et al., 2020), and immunomagnetic separation (Fujiwara-Nagata and Eguchi 2009). These methods have been more accurate in detecting *F. covae.* However, one major drawback is that these methods contain several procedural steps and take up to 48–56 h, which is a long time to respond to prevent a potential outbreak or treat an ongoing costly epizootic on a farm.

One objective of this study was to develop a rapid method to detect *F. covae* with flow cytometry (FCM). Flow cytometry has become a routine method for the microbiological analysis of freshwater and marine water samples (Gasol and Del Giorgio 2000; Cellamare et al., 2010). Fish pathogens and immune response have also been studied using FCM (Díaz-Rosales et al., 2006; Havixbeck et al., 2016). Flow cytometry can process multiple samples within minutes, making it preferable to other methods for enumeration studies. The FCM method uses light scattering and fluorescent properties of cells to make cell size and complexity measurements at population levels. Typically, in an FCM experiment, cells conjugated with a fluorochrome-labeled antibody are used to distinguish a cell population among many others in a sample. The enumeration procedure is completed within minutes. In this regard, FCM may prove to be a fast and reliable technique to characterize and enumerate *F. covae* populations from aquatic samples.

Bacterial disease outbreaks associated with intensive culture systems and environmental stressors pose a significant threat to freshwater aquaculture. As producers seek to incorporate intensive farming technologies such as split-pond systems (SPS; Stone et al., 2016), it becomes pertinent to investigate occurrences of stress-mediated pathogenesis in baitfish. Current investigation methods remain limited to the external examination of clinical signs and some time-consuming diagnostic laboratory procedures. A study was conducted to elicit pathophysiological responses of Golden Shiners held at two densities and two temperatures to an *F. covae* challenge. Also, an FCM protocol was developed for the rapid identification and enumeration of *F. covae* from aquatic samples.

## Materials and Methods

### Challenge System and Fish

The study was conducted in 40, 18-L Ultra-Low-Flow (ULF; Mitchell and Farmer 2010) tanks at the United States Department of Agriculture, Agricultural Research Service, Harry K. Dupree Stuttgart National Aquaculture Research Center, Stuttgart, Arkansas. Golden Shiners, mean weight (±S.D.) of 2.62 (±0.78) g each (observed negative for *F. covae*) were obtained from a commercial farm in Lonoke, Arkansas, United States.

### Experimental Design

Two trials were simultaneously conducted at 22°C and 28°C water temperature, each consisting of four treatments and five replicates per treatment. Fish were held at two densities (600 fish/m^3^ or 2,400 fish/m^3^) and subject to an *F. covae* challenge or not (control). The groups were: 600 fish/m^3^ and no *F. covae* challenge (6 fish/tank), 600 fish/m^3^ and *F. covae* challenge (6 fish/tank), 2,400 fish/m^3^ and no *F. covae* challenge (24 fish/tank), and 2,400 fish/m^3^ and *F. covae* challenge (24 fish/tank). Both studies followed the same procedure. Golden Shiners were held in a 400-L flow-through tank for acclimation to laboratory conditions. Fish were fed a commercial catfish diet to satiation once a day. After a 3-day acclimation period in the 400-L flow-through tank, fish were stocked into 40, 18-L ULF tanks containing 10 L of water. Aeration was provided to each tank with air stones. The tanks received filtered well water at about four exchanges per day or 30 ml/min from a ULF water delivery system (Mitchell and Farmer 2010). The water temperature in all tanks was maintained at either 22°C or 28°C. These temperatures were chosen to depict a temperature below the optimal range and one temperature within the optimum temperature range (25–30°C) for *F. covae* growth (Durborow et al., 1998; Plumb 1999; Roberts 2012). The water in the high-temperature treatment ULF tanks was heated using 50 W electric heaters (HPA-50), maintaining temperatures between 27.4°C and 28.5°C. The fish were no longer fed once moved to the challenge system.

After 3 days in the ULF-tanks, fish were subject to an *F. covae* challenge with the isolate MS-94-081 (genomovar II; Tekedar et al., 2012; Kumru et al., 2016). The frozen isolate was retrieved from the −80°C freezer and streaked on Ordal’s medium (Anacker and Ordal 1955). After 48 h, the isolate was dislodged from the Ordal’s medium with a sterile cotton swab and inoculated into 15 ml *F. covae* Growth Medium (FCGM; Farmer 2004). The inoculum suspension was incubated at 28°C for 24 h. The starter culture was then inoculated in 3 L of FCGM in three 1-L flasks. The flasks were incubated for 24 h at 28°C in an orbital shaker incubator at 200 rpm. The optical density of the isolate measured at 550 nm was 0.70, which equates nominally to 4.0 × 10^10^ CFU/ml. To allow full exposure of the fish to the bacteria, water flow was stopped to all tanks for 1 h after 100 ml of the cultured isolate was added to the tanks containing fish in the columnaris challenge groups. Water flow was then restored, and fish were observed for external clinical signs of columnaris. Mortalities were observed, counted, and removed 24 and 48 h post-challenge. Fish were considered moribund if they were unable to maintain neutral buoyancy. The study was ended 48 h after 50% mortality was achieved in at least one of the tanks. The remaining fish in tanks were euthanized using a lethal dose of MS-222 (250 mg/L), and *F. covae* samples were collected from tank water and fish tissue as described below.

### Water Flow and Quality

Water flow into each tank was measured on day 1 and 3. Water temperature and dissolved oxygen were measured daily in the tanks. Once daily in one tank for each treatment, total ammonia nitrogen, pH, and unionized ammonia were measured. Total ammonia nitrogen was measured using the salicylate method (Hach Co., Loveland, Colarado), pH was measured with a pH meter 49 (Denver Instrument, Denver, Colorado; Model UP-5), and unionized ammonia was measured following the method of Trussell (1972).

### DNA Extraction From Gill Tissue Samples for qPCR

Genomic DNA was extracted from gills, kidney, and spleen samples of two fish from each tank using the QIAcube DNA Purification System (QIAGEN, Valencia, CA, United States). Extraction was performed according to the manufacturer’s instructions. The extracted DNA was stored at −80°C until analysis by qPCR.

### *F. covae* Sample Collection for FCM and qPCR

Water samples from tanks were collected in 50 ml tubes and centrifuged at 4,136 × g for 10 min. After 48 ml of the supernatant was decanted from the tubes, the pellets were re-suspended in the same water and transferred to 2 ml centrifuge tubes. The samples were stored at 4°C until further analysis by qPCR or FCM.

### qPCR

Extracted DNA from fish gill, spleen, and kidney tissue (2.5 μl) and tank water (2.5 μl) were used for qPCR amplification with oligonucleotide primers targeting a 113-bp nucleotide region of the chondroitin A. C. lyase gene of *F*. *covae* (Panangala et al., 2007). The primer sequence was as follows FcFd (forward) CCT​GTA​CCT​AAT​TGG​GGA​AAA​GAG​G, FcRs (reverse) GCG​GTT​ATG​GCC​TTG​TTT​ATC​ATA​GA and probe ACA​ACA​ATG​ATT​TTG​CAG​GAG​GAG​TAT​CTG​ATG​GG. This procedure confirmed the identity of isolates as *F. covae* and quantified bacterial DNA from the samples. The TaqMan-based real-time PCR amplification reaction mixture consisted of 1X TaqMan™ Universal PCR Master Mix containing AmpliTaq Gold DNA polymerase, each dNTP, a passive reference dye (ROX) and optimizing buffer components (Applied Biosystems, Foster City, CA, United States), 400 nM each of forward (FcFp) and reverse (FcRp) primers, 200 nM labeled probe, 5 µL template DNA and sterile molecular grade water to make a final reaction volume of 25 µL. The reactions were performed in triplicate with the cycling parameters set as follows: an initial DNA denaturation and AmpliTaq activation at 95°C for 10 min, followed by 45 cycles of 95°C for 15 s and 60°C for 1 min (Applied Biosystems, StepOne Plus real-time PCR Thermo Scientific).

### Flow Cytometry

Anti-*F. covae*, IgG fraction monoclonal antibodies, (clone 4H10/F12), (Austral Biologicals, San Ramon, CA, United States; Catalog # FM-070AZ-5) were labeled with Fluorescein (Lightning-Link^®^ Fluorescein Conjugation Kit, Innova Biosciences, Catalog # 707-0015) according to the manufacturer’s standard protocols. The resulting fluorescein-labeled antibody conjugate (2 μl) was then mixed with centrifuged tank water samples (100 μl) and vortexed for 30 s before being analyzed with a BD Accuri Flow Cytometer (Becton, Dickinson, and Company, Franklin Lakes, NJ, United States). Using the FCM to enumerate bacteria, 100 µL samples were analyzed at the “high” rate (100 µL/min) in triplicate. The threshold was set on the green fluorescence (FL1-H). Count data and fluorescence measurements were obtained with BD Accuri C6 software and FlowJo (FlowJo LLC, Becton, Dickinson, and Company, Franklin Lakes, NJ, United States) to generate cytograms. Populations of *F. covae* were identified on cytograms obtained from forward scatter (FSC-H) vs. side scatter (SSC-H) and confirmed by FL1-H vs. SSC-H density plots.

### Cortisol Analysis

At the end of the study, two fish from each treatment group were euthanized with MS-222 and stored at −80°C for cortisol analysis. A whole-body cortisol analysis was conducted on a pooled sample of two individuals per treatment, according to Sink et al. (2007b).

### Histology

Gill tissue samples from fish subject to columnaris challenge and control treatments were fixed, sectioned, and stained according to standard hematoxylin and eosin protocol (Bancroft and Stevens 1995) for determining the pathological changes in gill structure from columnaris infection.

### Statistical Analysis

To determine differences in water quality parameters among treatments, a generalized linear model (GLM) was used for each water quality parameter. Based on normality testing, the conducted GLMs were either parametric (analysis of variance; ANOVA) or non-parametric (Kruskal-Wallis test). To test for the main effects of challenge (control and bacteria), temperature (22°C and 28°C), and density (600 and 2,400 fish/m^3^); and their interactions on each tested variable, a generalized linear mixed model (GLMM) was used. When normality assumption was violated, GLMMs were applied on rank transformed data. In all linear model analyses, if there were significant differences, post-hoc analyses were performed using the Tukey’s Studentized Range—HSD [or Dwass-Steel-Critchlow-Fligner (DSCF) for rank data].

Kaplan-Meier survival analyses were performed for estimation of median survival times and survival probabilities of Golden Shiners 48 h after challenge based on the challenge, temperature, and density. Log-rank tests were used to compare the survival probability between challenge groups (control versus bacteria), temperatures, and densities.

Counts of *F. covae* in water samples determined by FCM and qPCR were compared using paired sample t-test if the data were normally distributed and Wilcoxon signed-rank test if the data were not normally distributed. The relationship between both counts was examined using Spearman’s rank-order correlation test (data did not exhibit a normal distribution). Moreover, Bland–Altman analysis was performed to assess the agreement in cell counts determined by the two quantitative detection methods (FCM and qPCR) in the same water samples.

Statistical significance was set at *p* < 0.05. Levene’s test was used to evaluate the homogeneity of variances (homoscedasticity), and the Shapiro–Wilk test was utilized for normality analysis of the variables. Bacterial counts were logarithmic transformed (base 10) and presented as log (count of *F. covae* + 1)/ml. All normally distributed data were presented as the mean ± standard error of the mean (*SE*) while non-normally distributed data were presented as median ± interquartile range (*IQR* = percentile 75%−percentile 25%). Data management and all statistical analyses were performed with SAS^®^ version 9.4 (SAS 2013). All graphs were constructed in SigmaPlot^®^ software (version 14.5; Systat Software Inc., San Jose, CA, United States).

## Results

Weight of Golden Shiners used in this study ranged from 1.16 to 5.78 g (2.62 ± 0.11 g; *n* = 50). While their body length ranged between 5.10 and 8.40 cm (6.64 ± 0.08 cm). After 24 h of exposure, fish in the challenged groups started displaying clinical signs typical of columnaris disease, which included depigmentation, caudal and dorsal fin margins erosions, and characteristic “saddleback” lesions (Noga, 1996). As the disease progressed, infected tissues became necrotic and eventually resulted in fish mortality.

### Water Flow and Quality

Concentrations of unionized ammonia (NH_3_) and pH did not differ among treatments (Table 1). Significant differences occurred in water flow rates, the percentage of NH_3_, total ammonia nitrogen (NH_4_), water temperature, and dissolved oxygen (DO; Table 1). Flow rates for the control group at 22°C stocked with 2,400 fish/m^3^ were significantly lower than the control groups of 600 and 2,400 fish/m^3^ at 28°C, the bacterial group of 600 fish/m^3^ at 22°C and 2,400 fish/m^3^ at 28°C (Table 1). The percent of unionized ammonia in the control tanks stocked at 2,400 fish/m^3^ was significantly lower than the NH_3_ in the control tanks stocked at 2,400 fish/m^3^ and the bacterial tank stocked at 600 fish/m^3^, both of which were at 28°C (Table 1). Concentrations of NH_4_ of fish stocked in control tanks and bacterial tanks stocked at 600 fish/m^3^ at 22°C were significantly lower than all tanks stocked at 2,400 fish/m^3^ (Table 1). As expected, the lower water temperature was significantly different within control and bacterial groups stocked at both densities compared to those maintained at the higher temperature (Table 1). Similarly, DO was higher at the lower temperature compared to the higher temperatures for all groups (Table 1).

**TABLE 1:** Water quality [number of measurements (*n*), mean, standard error of the mean (*SE*), minimum measurement (min), and maximum measurement (max)] measured in tanks of eight treatments. Golden Shiners were subjected to low (600 fish/m^3^) or high (2,400 fish/m^3^) densities at low (22°C) or high (28°C) temperature treatments. Treatments included challenge with *F. covae* (Bacteria) or no exposure to *F. covae* (Control). Based on normality testing, tests used were either analysis of variance (test statistics = *F*_df1,df2_) or Kruskal-Wallis [test statistics = χ^2^_(df)_]. Within each parameter, means with different lowercase superscript letters are significantly different at *p* < 0.05 if **bold**.

Water quality parameter	Density (fish/m^3^)	Control				Bacteria				Overall	
		22°C		28°C		22°C		28°C			
		*n*	Mean ± *SE* (min–max)	*n*	Mean ± *SE* (min–max)	*n*	Mean ± *SE* (min–max)	*n*	Mean ± *SE* (min–max)	Mean ± *SE* ^a^ (min–max)	Test statistics (*p*-value)^b^
pH	600	2	8.49 ± 0.04	1	8.34	2	8.48 ± 0.01	2	8.45 ± 0.07	8.42 ± 0.03	*F*_7,6_ = 2.63
	2,400	2	8.26 ± 0.08	1	8.50	2	8.46 ± 0.03	2	8.37 ± 0.04		
Water flow (ml/min)	600	10	28.40^a^ ^,^ ^b^ ± 0.48	8	30.00^a^ ± 0.50	10	29.60^a^ ± 0.58	10	29.20^a^ ^,^ ^b^ ± 0.63	29.06 ± 0.25	*F*_7,64_ = 3.33
	2,400	6	26.50^b^ ± 0.96	8	29.75^a^ ± 0.77	10	28.00^a^ ^,^ ^b^ ± 0.65	10	30.30^a^ ± 0.60		
Unionized-NH_3_ (mg/L)	600	2	0.02 ± 0.00	1	0.04	2	0.02 ± 0.00	2	0.08 ± 0.04	0.06 ± 0.01	*F*_7,6_ = 3.99
	2,400	2	0.05 ± 0.01	1	0.16	2	0.08 ± 0.01	2	0.10 ± 0.01		
NH_3_ (%)	600	2	12.85^a^ ^,^ ^b^ ± 0.95	1	13.50^a^ ^,^ ^b^	2	12.50^a^ ^,^ ^b^ ± 0.20	2	16.65^a^ ± 2.35	13.11 ± 0.83	*F*_7,6_ = 5.18
	2,400	2	8.05^b^ ± 1.25	1	18.20^a^	2	11.95^a^ ^,^ ^b^ ± 0.45	2	13.90^a^ ^,^ ^b^ ± 0.80		
NH_4_ (mg/L)^c^	600	7	0.13^b^ ± 0.07	5	0.26^a^ ^,^ ^b^ ± 0.24	7	0.14^b^ ± 0.05	6	0.41^a^ ^,^ ^b^ ± 0.53	0.39 ± 0.57	χ^2^_(7)_ = 22.94
	2,400	4	0.64^a^ ± 0.10	4	0.62^a^ ± 0.20	6	0.72^a^ ± 0.11	5	0.71^a^ ± 0.27		
Water temperature (°C)^c^	600	15	22.40^b^ ± 0.30	12	28.10^a^ ± 0.50	15	22.30^b^ ± 0.40	15	28.10^a^ ± 0.30	25.05 ± 5.65	χ^2^_(7)_ = 82.15
	2,400	9	22.40^b^ ± 0.40	12	28.00^a^ ± 0.30	15	22.30^b^ ± 0.50	15	28.00^a^ ± 0.40		
Dissolved oxygen (mg/L)^c^	600	15	8.19^a^ ± 0.49	12	7.47^b^ ± 0.24	15	8.31^a^ ± 0.75	15	7.56^b^ ± 0.18	7.63 ± 0.77	χ^2^_(7)_ = 52.83
	2,400	9	8.04^a^ ± 0.31	12	7.48^b^ ± 0.23	15	8.24^a^ ± 0.72	15	7.36^b^ ± 0.25		

a Medians and means in “overall” column represent all recorded measurements for a given water quality parameter (all eight treatment groups).

b Statistical comparisons among eight treatment groups for a given water quality parameter.

c Data are presented as median ± interquartile range (25th percentile–75th percentile) and (min–max) because normality assumption was violated.

### Survival

**FIGURE 1:**
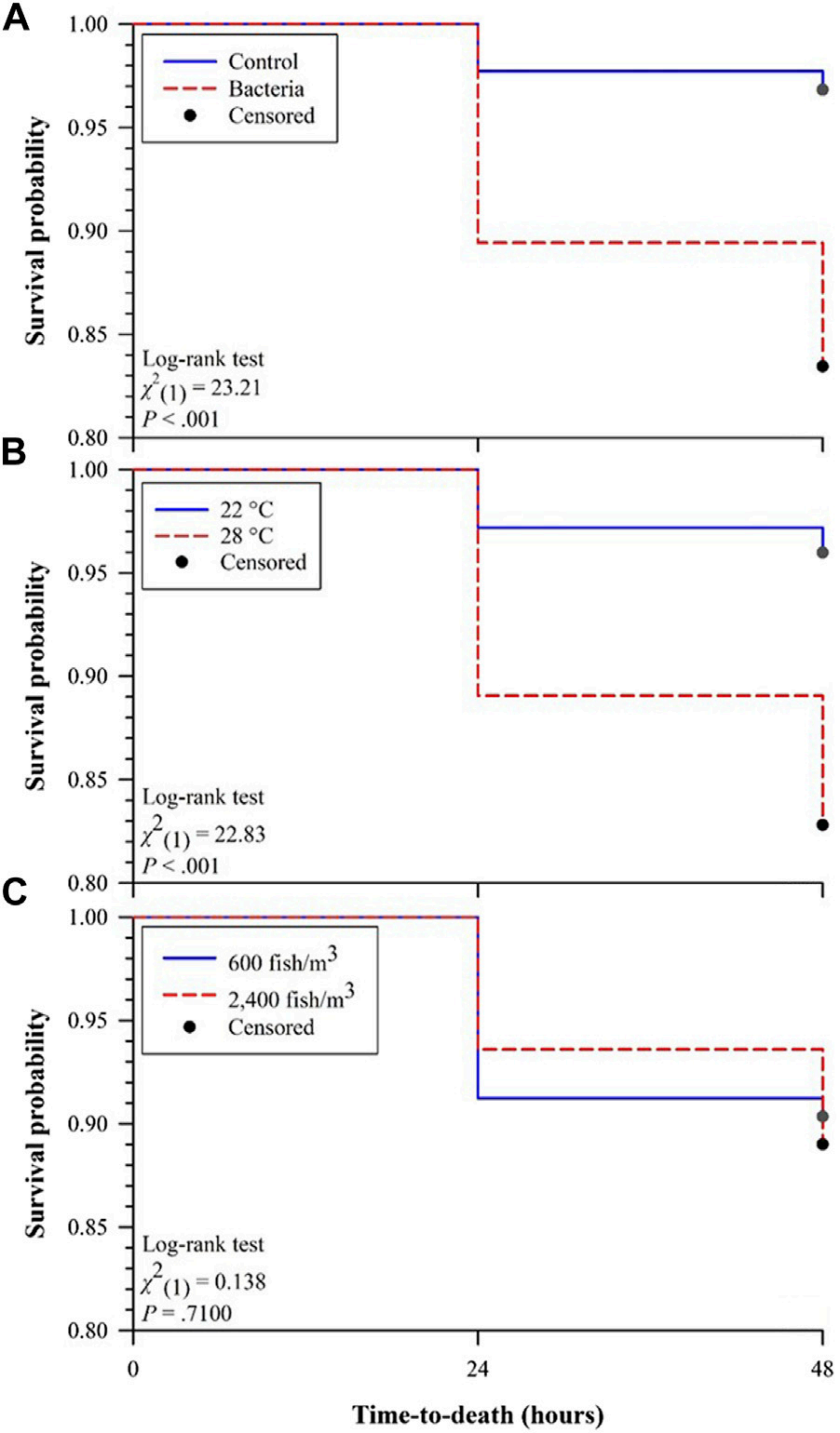
Kaplan-Meier survival curves for effects of **(A)** challenge (control versus bacteria), **(B)** water temperature (22°C versus 28°C), and **(C)** fish density (600 versus 2,400 fish/m^3^) on survival probabilities of Golden Shiners 48 h after challenge.

Comparison of Kaplan-Meier survival curves (Figure 1) demonstrated that survival probability of Golden Shiners challenged with *F. covae* (bacteria) were significantly lower than those without exposure (control; Figure 1A). Golden Shiners held at 22°C had significantly higher survival as compared to those held at 28°C (Figure 1B) while both fish densities (600 and 2,400 fish/m^3^) did not differ in survival (Figure 1C).

Examination of the two-way interaction of challenge × density, survival percent across the study was significantly different, but did not differ in the other interactions (Table 2). Survival percent of control tanks stocked with 600 fish/m^3^ were significantly higher than those of bacterial tanks stocked with 2,400 fish/m^3^ (Table 3). Survival percent after the 48 h challenge study, showed a significant difference in the challenge × temperature, but did not differ in the other interactions (Table 2). Survival of the control groups at 22°C was significantly higher than the bacterial groups at 28°C (Table 3).

**TABLE 2:** Statistical analysis results (test statistics, degrees of freedom, and *p*-value) of two-way interactions (groups = 4) and three-way interactions (groups = 8) between the three main effect variables (challenge, temperature, and density) for survival, fish qPCR, water qPCR, and flow cytometer bacterial count of *F. covae* in water. Significant results at *p* < 0.05 if **bold**.

Parameters	Challenge × temperature		Challenge × density		Temperature × density		Challenge × temperature × density	
	χ^2^_(3)_	*p-*value	χ^2^_(3)_	*p-*value	χ^2^_(3)_	*p-*value	χ^2^_(7)_	*p-*value
Survival (%) across the study	7.30	0.0628	8.31	**0.0401**	4.13	0.2480	12.49	0.0856
Survival (%) 48 h after challenge	7.87	**0.0488**	7.67	0.0534	3.49	0.3217	11.35	0.1239
Water flow cytometer [log (count + 1)/ml]	25.73	<0**.0001**	26.55	<0**.0001**	2.14	0.5429	27.41	**0.0003**
Water qPCR [log (count + 1)/ml]	30.99	<0**.0001**	28.47	<0**.0001**	1.49	0.6837	31.03	<0**.0001**
Fish qPCR [log (count + 1)/ml]	7.36	0.0613	6.87	0.0762	0.49	0.9205	8.32	0.3051

**TABLE 3:** Median, interquartile range (*IQR*), minimum measurement (min), and maximum measurement (max) of survival % and log (count of *F. covae* + 1)/ml of fish qPCR, water qPCR, and flow cytometer bacterial count in water for challenge-temperature-density treatment groups (*n* = 8).

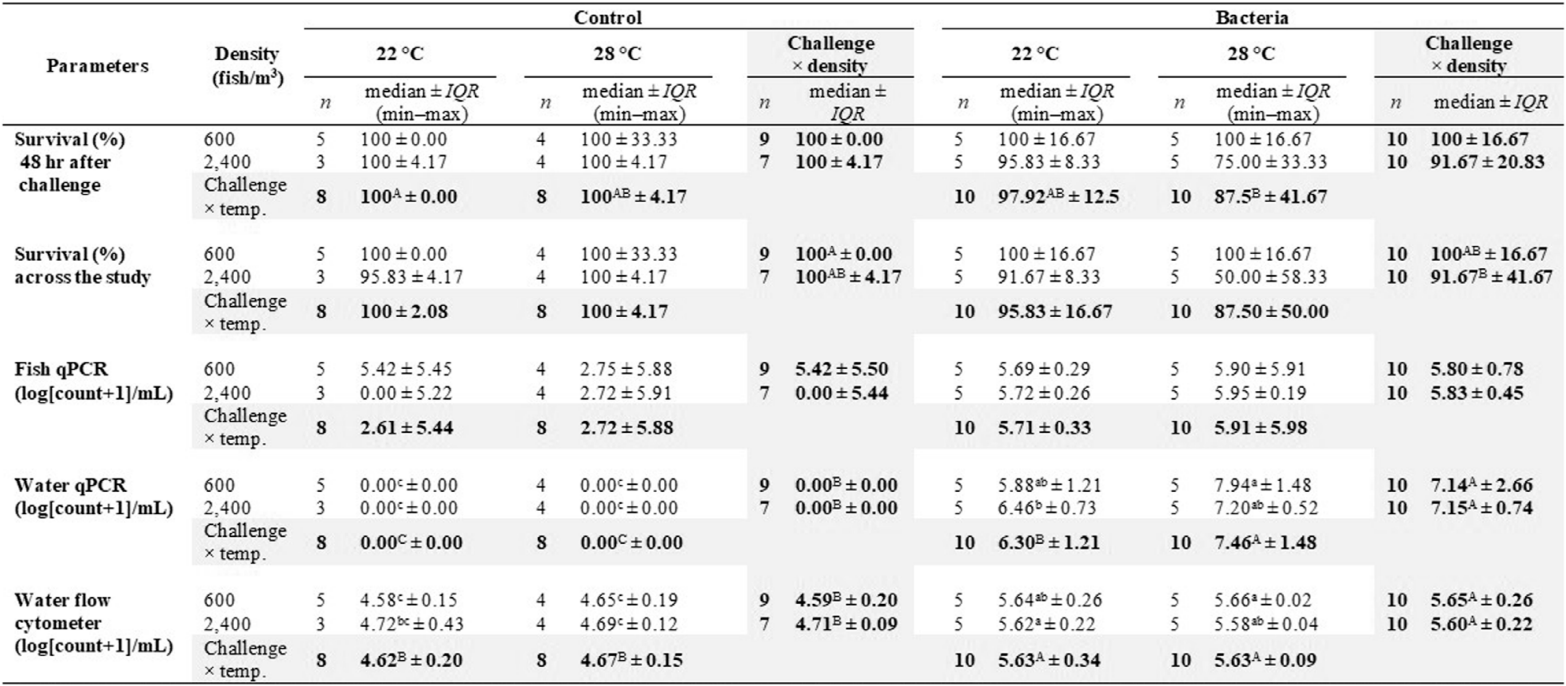

Within each parameter, mean values not sharing lowercase superscript letters indicate significant differences among treatments at *p* < 0.05. The “challenge × temp.” rows represent means over both fish densities within each challenge-temperature group (*n* = 4) for each parameter. Within the same “challenge × temp.” row, means with different uppercase superscript letters are significantly different at *p* < 0.05. The “challenge × density” columns represent means over both temperatures within each challenge-density group (*n* = 4) for each parameter. Within the same parameter, “challenge × temp.” means with different uppercase superscript letters are significantly different at *p* < 0.05, if **bold**.

**FIGURE 2:**
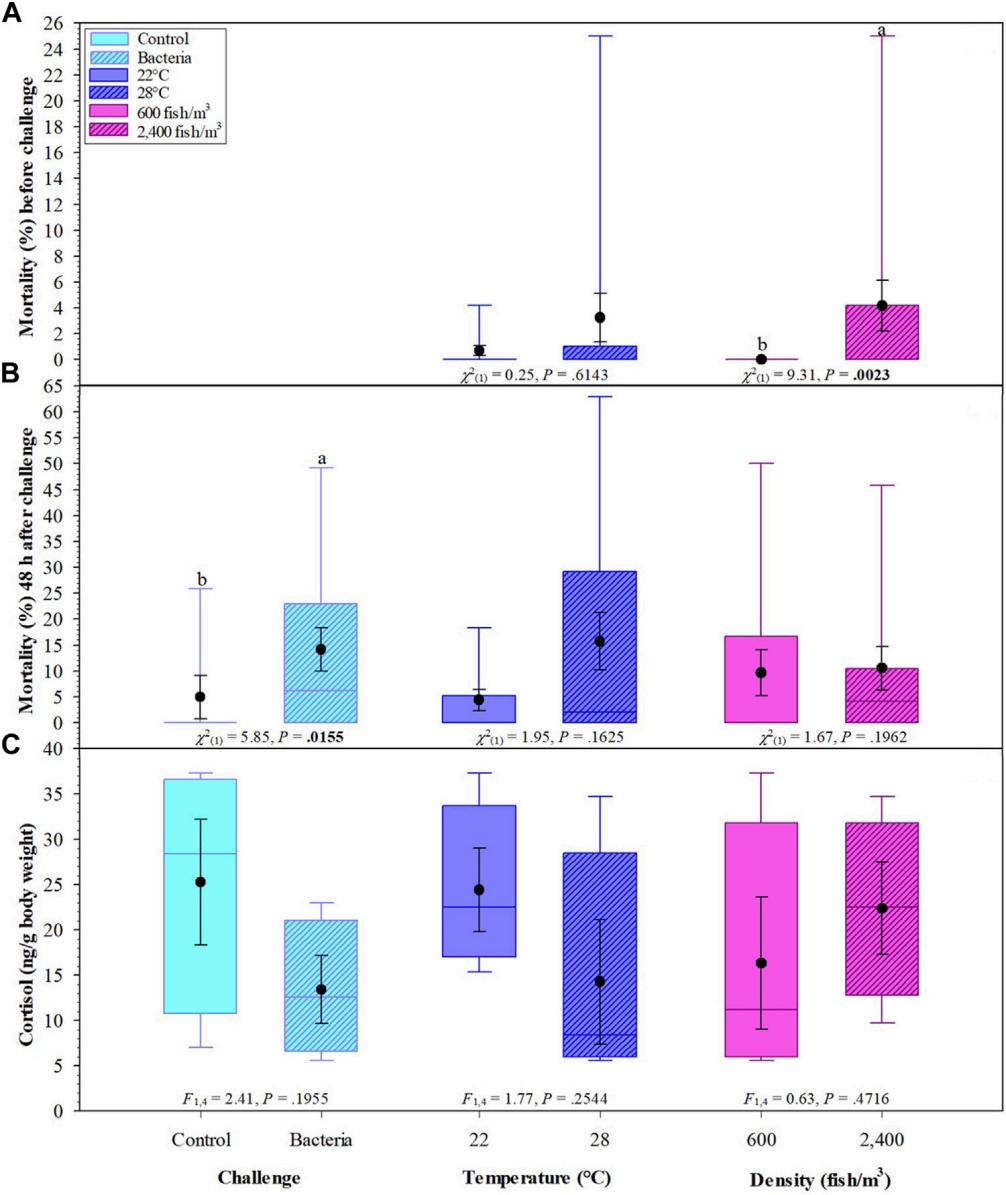
Effects of challenge, temperatures, and fish density of Golden Shiners on mortality (%) before challenge **(A)**, after challenge **(B)** and whole-body cortisol (ng/g body weight) **(C)**. Golden Shiners were subjected to low (600 fish/m^3^) or high (2,400 fish/m^3^) densities at low (22°C) or high (28°C) water temperature treatments. Treatments included challenge with *F. covae* (Bacteria) or no exposure to *F. covae* (Control). Within each box plot, vertical line indicates the median, black dot indicates the mean, and error bars around the black dot represent standard error of the mean.

There was a significant difference in mortality before the challenge with tanks stocked at 600 fish/m^3^ significantly lower than those stocked at 2,400 fish/m^3^ (Figure 2A). Mortality percentages 48 h after bacterial challenge were lower in the control groups vs. the bacterial groups (Figure 2B).

### Enumerating Bacteria Using FCM and qPCR

**FIGURE 3:**
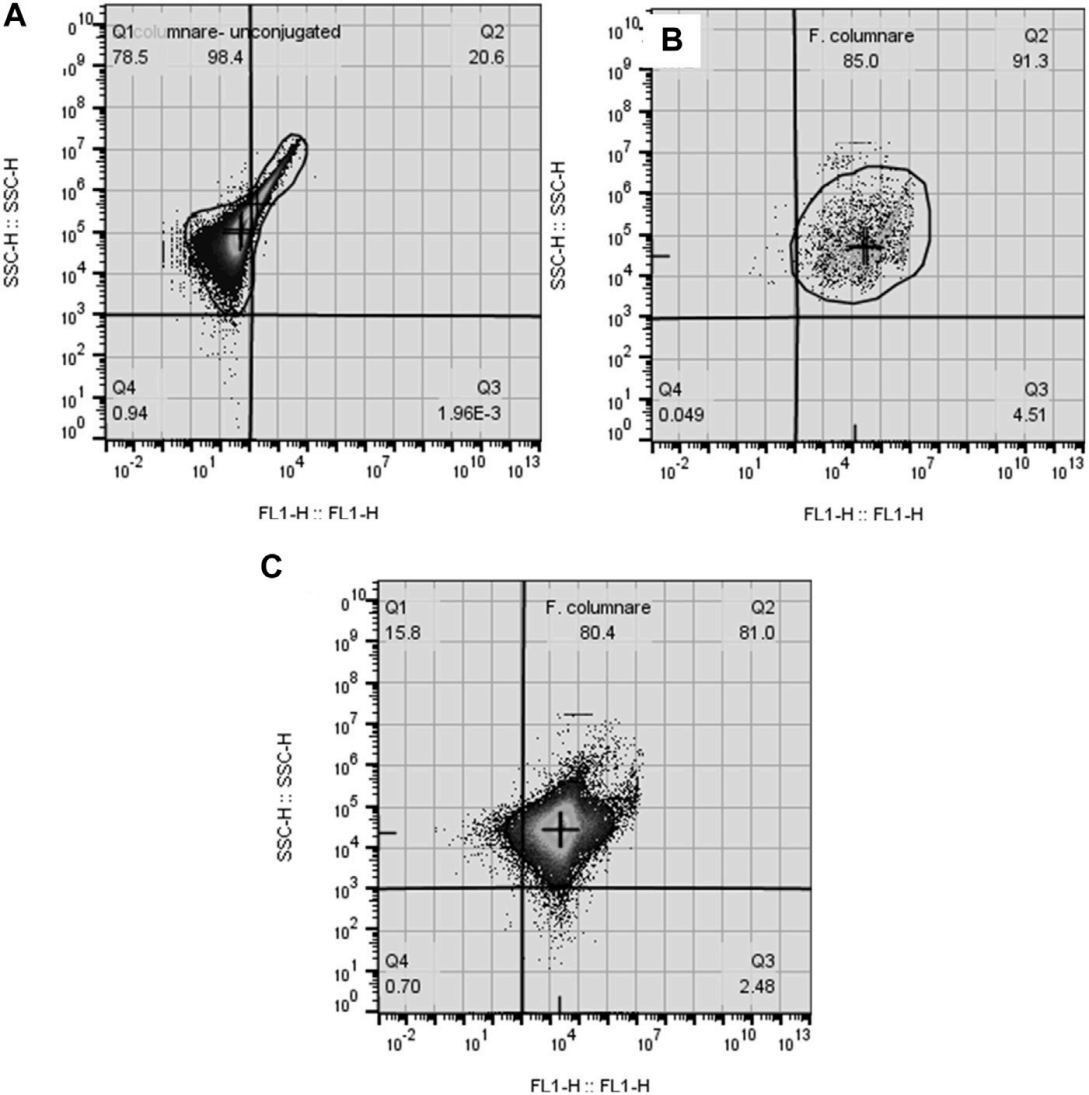
Cytograms depicting fluorescence density plot (FL1-H vs. SSC-H) of **(A)** unconjugated *F. covae* cells obtained from bacterial cell culture and *F*. *covae* cell samples conjugated with fluorescein labelled antibodies from **(B)** control tanks containing well water and **(C)** columnaris challenge tanks where fish were exposed to *F. covae*. Plots **(B,C)** show a shift in fluorescence to the right side of the plot indicating fluorescence of the conjugated antibody attached to *F. covae* cells.

**FIGURE 4:**
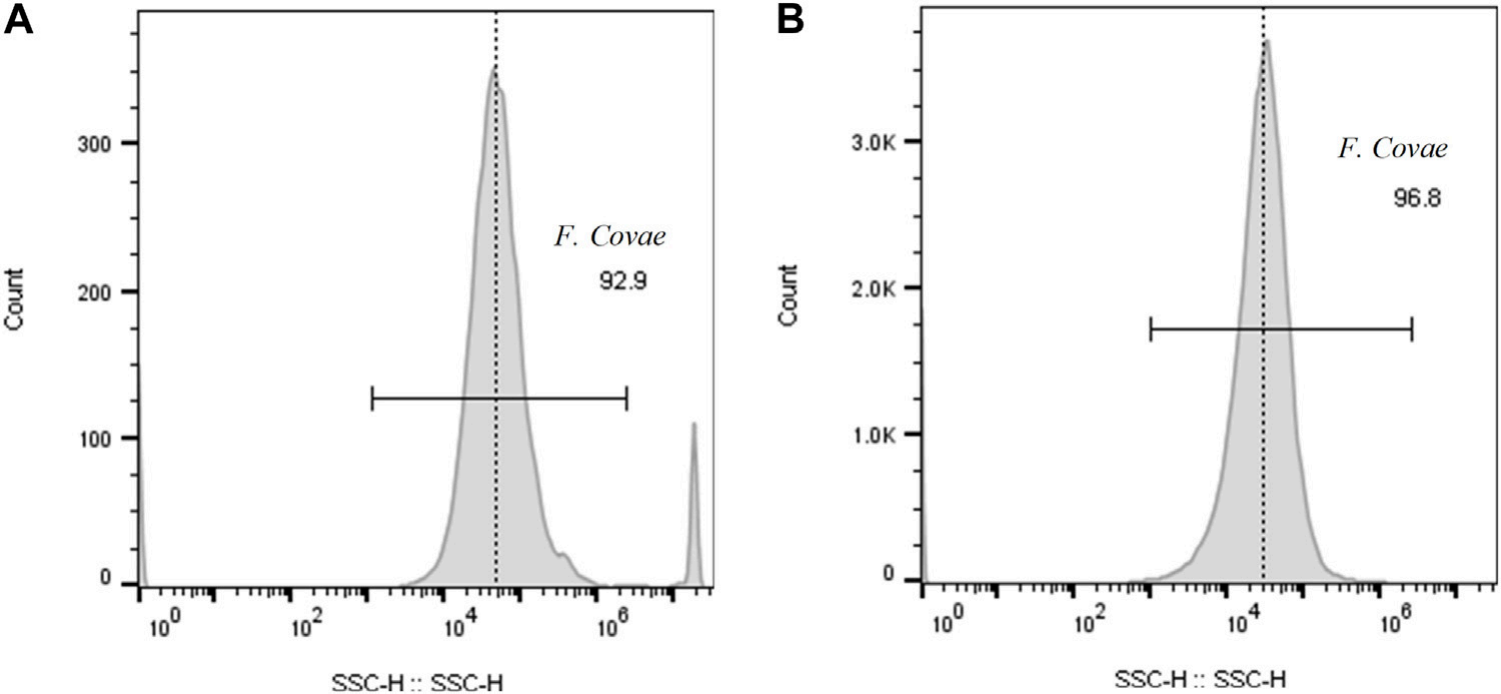
Histogram plots of *F. covae* cell counts obtained from **(A)** control tank water samples and **(B)** tanks exposed to *F. covae* challenge. Note the scale on the y-axis in **(B)** is an order of magnitude higher than **(A)**.

Over 90% of the events enumerated in the water samples were *F. covae* cells, as evidenced by the SSC-H histograms (Figure 3) and FL1-H density plots (Figure 4). Density plots obtained from fluorescein-labeled antibody conjugated *F. covae* cells showed a right shift in fluorescence in the FL1 region (530 ± 15 nm) compared to the unconjugated cells (Figure 4).

**FIGURE 5:**
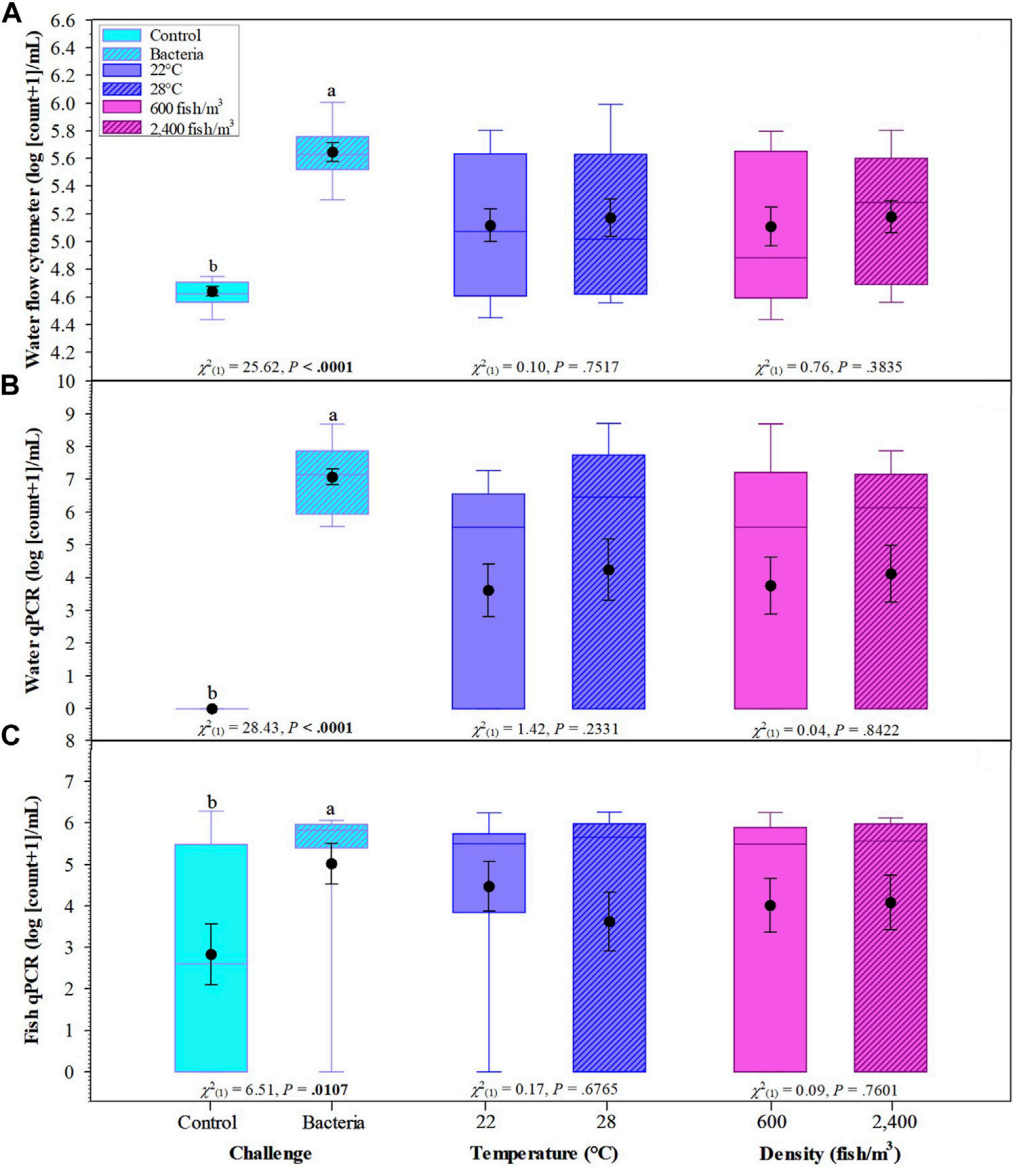
Effects of challenge, temperatures, and fish density of Golden Shiners on flow cytometer bacterial count in water **(A)**, water qPCR **(B)**, and fish qPCR **(C)**. Golden Shiners were subjected to low (600 fish/m^3^) or high (2,400 fish/m^3^) densities at low (22°C) or high (28°C) temperature treatments. Treatments included challenge with *F. covae* (Bacteria) or no exposure to *F. covae* (Control). Within each box plot, vertical line indicates the median, black dot indicates the mean, and error bars around the black dot represent standard error of the mean. Data with different lowercase letters within same variable differ significantly at *p* < 0.05 if **bold**.

Overall, *F. covae* counts obtained by FCM were significantly higher in the columnaris challenge treatments than in the control treatments (Figure 5A). There were no significant differences in bacterial counts in the density or temperature treatments.

Bacterial counts/ml obtained from water samples determined by qPCR were significantly higher in the columnaris challenge treatment groups than the control treatments. There were no significant differences between bacterial counts/ml obtained from water samples in the density or temperature treatments (Figure 5B). Similar results were obtained for fish bacterial counts *via* qPCR with control fish having lower bacterial counts compared to fish challenged with *F. covae* (Figure 5C).

There were significant differences between water bacterial counts determined by FCM when examining the interaction of challenge × temperature and challenge × density as well as the three-way interaction of challenge × temperature × density (Tables 2 and 3).

### Validation of FCM With qPCR for Tank Water

**FIGURE 6:**
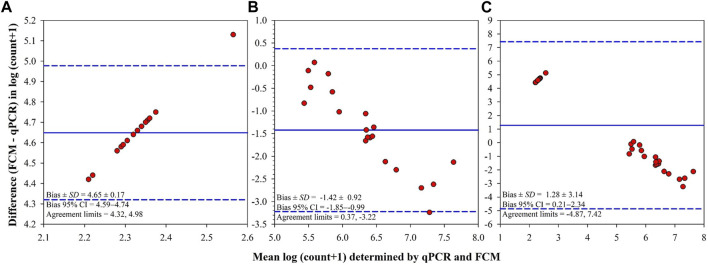
Bland-Altman summaries for *F. covae* cell counts determined by quantitative PCR (qPCR) and flow cytometry (FCM) in the same water samples obtained from control tanks **(A)**, tanks exposed to *F. covae* challenge **(B)**, and all tanks **(C)**. The Bland-Altman summaries plot the difference in the measure [log (count of *F. covae* + 1)] between two determination methods (FCM–qPCR; y-axis) versus the mean of the measure determined by the two methods for each subject (x-axis). The overall mean differences (bias; solid lines) and limits of agreement (mean difference ±1.96 standard deviation of the differences; dashed lines) are also shown on the plots.

There was a significant positive correlation between bacterial counts determined by qPCR and those determined by FCM in all tanks (Spearman’s rank-order: *r*_s_(36) = 0.88, *p* < 0.0001) and challenge tanks (Spearman’s rank-order: *r*_s_(20) = 0.60, *p* = 0.0063). The Bland–Altman plots support these strong correlations, however, there were differences between the counts determined by qPCR and FCM as shown in the Bland–Altman difference plots (Figure 6). In control tanks, limits of agreement (LOA) did not include the Bland–Altman identity line (difference = 0.0; Figure 6A). All Bland–Altman plots show that most points fall within LOA and show a proportional constant error hence the variability of differences between both determination methods increased as the magnitude of the measure increased (Figure 6).

**FIGURE 7:**
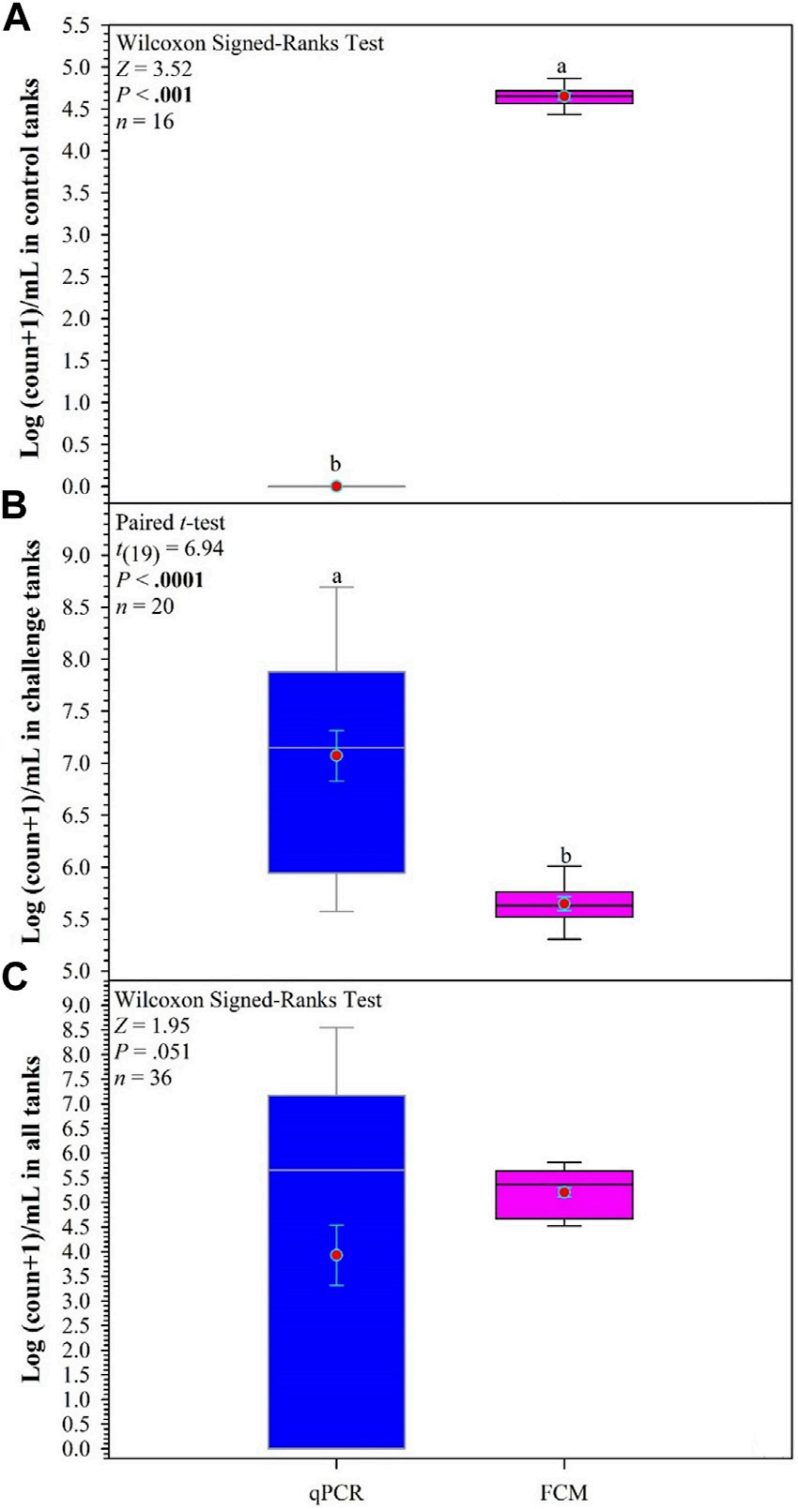
Comparisons between quantitative PCR (qPCR) and flow cytometry (FCM) to determine count of *F. covae* in water samples obtained from control tanks **(A)**, challenge tanks **(B)**, and all tanks **(C)**. Within each box plot, vertical line indicates the median, red dot indicates the mean, and error bars around the red dot represent standard error of the mean. Data with different lowercase letters within same variable differ significantly at *p* < 0.05 if **bold**.

The comparison of bacterial counts determined by qPCR and FCM in control tanks indicates a significant difference in the counts, with the FCM count higher than the qPCR counts (Z = 3.52, *p* < 0.001; Figure 7A). However, in the challenge tanks, the results are reversed with higher bacterial counts determined by qPCR [*t*_(19)_ = 6.94, *p* < 0.0001; Figure 7B]. The overall counts in all tanks show no difference in the counts obtained, but the range obtained with qPCR is greater, although not significantly (Figure 7C).

### Cortisol Analysis

Across all treatments, whole-body cortisol ranged from 5.60 to 37.30 ng/g body weight with no significant differences in fish cortisol concentrations between challenge, temperature, or density groups (Figure 2C).

### Histology

**FIGURE 8:**
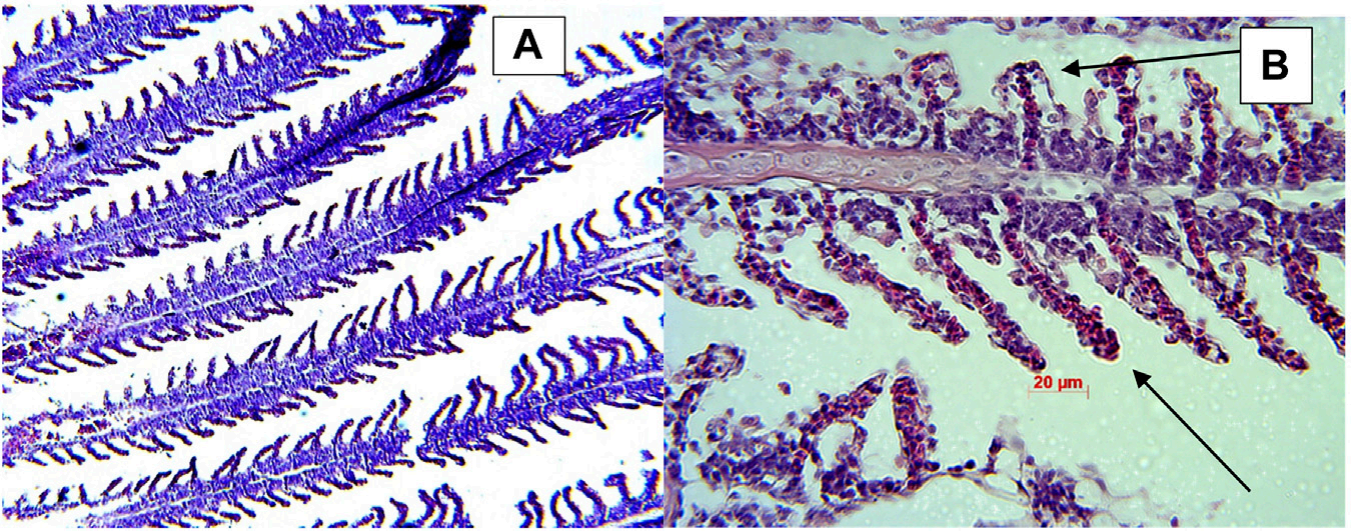
Histology samples of gill tissue showing retention of normal gill structure in control fish **(A)** and clubbed gills indicated by black arrows columnaris infected fish **(B)**.

Microscopic examination of the affected gill tissue revealed characteristic columnaris infection signs—the disappearance of the typical structure and clubbing of primary and secondary filaments in fish exposed to the bacterial challenge. Samples from control groups revealed a typical gill structure (Figure 8).

## Discussion

Although significant differences occurred in some of the water quality parameters (NH_3,_ NH_4_, DO and temperature) the values obtained were within acceptable limits for culture of the Golden Shiner (Stone et al., 2016) and was likely not a cause of the mortality observed in this study.

Survival in Golden Shiners was not affected by rearing density (6,00,000–24,00,000 fish/ha). Golden Shiners are gregarious and a shoaling species (Pitcher 1983) that can tolerate high densities and therefore, is the reason higher densities did not affect survival. However, survival was significantly impacted in high-density groups exposed to the challenge. This result supports the view that higher rearing densities can lead to increased transmission of disease. A 6°C increase in temperature from 22°C to 28°C led to a significant decrease in survival when fish were challenged. Suomalainen et al. (2005) noted that high temperature (+23°C) and high rearing density increased mortality in rainbow trout. As Golden Shiners are a warm water species, higher water temperatures within the culture range may not affect Golden Shiners survival, but it could increase infection severity, as evidenced by the significantly higher bacterial count by qPCR from gill tissue.

Cortisol measurements showed no variation among the treatment groups suggesting the densities and temperature treatments in the study were well within the range used for rearing Golden Shiners. The average cortisol measurement (19.3 ng/g) were comparable to those observed by Melandri et al. (2008) 12–15 ng/g for Golden Shiners under non-stressed conditions. Stressed Golden Shiners show elevated cortisol measurements as observed with handling stress (46.5 ng/g; Sink et al., 2007a) and acute 30-min confinement stress (51.2 ng/g; Sink et al., 2008).

It is essential to note the difference in virulence among isolates of *F. covae* bacteria (Pulkkinen et al., 2010; Kumru et al., 2017) to different species. The isolate MS-94-081 is a highly virulent obligate pathogen in catfish (Soto et al., 2008; Kumru et al., 2017). However, this isolate is not as virulent in Golden Shiners, as it did not result in 100% mortality even at the end of 48 h challenge. Additionally, Golden Shiners were held in ULF flow-through tanks, which may have reduced the contact time between the pathogen and the host tissue. An earlier study noted that mortality rates of elvers (Order Anguilliformes) were reduced by half in tanks with running water compared with those held in static water (Kuo et al., 1981).

Although it appears that bacterial enumerations by FCM were comparable to counts by qPCR, it is important to examine the bias produced in the Bland-Altman plots. In this study the bias was significant because the line of equity (line at y = 0) is not within the confidence intervals of the mean difference. However, clinically, if the presence or absence of bacteria is the acceptable criteria then the FCM and qPCR methods could be used interchangeably. Bacterial counts by PCR were higher than the FCM enumerations. This was expected as counts by PCR are estimated by DNA concentrations in the form of live and dead cells. Furthermore, qPCR is highly sensitive; as few as three bacteria/ml can be detected from samples (Panagala et al., 2007). On the other hand, to obtain meaningful data, FCM samples had to be concentrated. FCM measurements are affected quantitatively and qualitatively by the fluorochrome used and storage temperature (Jacquet et al., 2013). This could explain the discrepancy between the counts obtained by the two methods. FCM and qPCR produced strongly correlated results; this was expected as both quantify target populations.

## Conclusion

Golden Shiners raised at increased densities and temperatures in intensive rearing aquaculture systems, even within the normal range for growth of this species does not effect the stress response. Higher temperatures increase the mortality rate in fish maintained at 2,400 fish/m^3^ and challenged with *F. covae.* To obtain counts by FCM, a fluorescein labelled monoclonal antibody was conjugated with the bacterial cells and analyzed in a flow cytometer. Bacterial counts by FCM showed a strong correlation with bacterial DNA numbers by PCR in the water samples. FCM is a fast and reliable method for counting *F. covae* from experimental tank water samples. However, the reliability of the method needs to be tested for pond water samples containing other flora.

## Data Availability

The raw data supporting the conclusion of this article will be made available by the authors, without undue reservation.
